# Metastatic sclerosing epithelioid fibrosarcoma in an adult male from a tertiary care centre in India: a case report

**DOI:** 10.3332/ecancer.2022.1446

**Published:** 2022-09-20

**Authors:** Akhil Santhosh, Jay Mehta, Adarsh Barwad, Shamim A Shamim, Sameer Rastogi

**Affiliations:** 1 Department of Medical Oncology, BRA IRCH, All India Institute of Medical Sciences, New Delhi 110029, India; 2 Department of Pathology, All India Institute of Medical Sciences, New Delhi 110029, India; 3 Department of Nuclear Medicine, All India Institute of Medical Sciences, New Delhi 110029, India

**Keywords:** SEF, low-grade fibromyxoid sarcoma, MUC 4, EWSR1, FUS-CREB3L1

## Abstract

**Background:**

Sclerosing epithelioid fibrosarcoma (SEF) is an extremely rare subtype of soft tissue sarcoma and the data from India is sparse. It is an unusual variant of fibrosarcoma that commonly arises in the soft tissues of the limb, head and neck, trunk and occasionally in the visceral organs and bones. This entity is commonly reported in the middle age group, men and women alike. Pathological clinchers include MUC 4 (Mucin 4, cell surface associated) positivity by immunohistochemistry, FUS-CREB3L1 fusion and EWSR1 rearrangement. This disease is notoriously known for its local recurrence and metastatic spread. Response to systemic therapy is poor and relapses are frequent. The role of targeted and immunotherapy is not well defined.

**Case presentation:**

Here we report a 46-year-old gentleman who presented to the Sarcoma Medical Oncology Clinic in our centre. He had primary involvement of right pubic bone with metastasis to liver, lung and diffuse lytic bony lesions. His diagnosis was reviewed multiple times before coming to final diagnosis of SEF. His molecular test for EWSR1 rearrangement was positive by fluorescence in-situ hybridisation. He did not respond to palliative doxorubicin, pazopanib and gemcitabine and docetaxel.

**Conclusion:**

Through this case report, we would like to highlight the rarity of this sarcoma, its classical pathological features, its close relationship to low-grade fibromyxoid sarcoma and the limited therapeutic options available. Hence, there is a need for further research in this entity.

## Introduction

Sclerosing epithelioid fibrosarcoma(SEF) is one of the rare varieties of fibrosarcoma which affects men and women alike in their middle ages [1]. This rare entity belongs to the group of fibrosarcomas which also includes fibromyxoid sarcomas and hyalinising spindle cell tumour with giant rosettes. This disease was initially reported by Meis-Kindlom and colleagues in 1995, as a distinct tumour of the soft tissue and deep musculature , with frequent involvement of the adjacent fascia and/or periosteum [2].

The median age is 45 years, with a slight male preponderance. These tumours mostly occur in the lower extremities and limb girdles, trunk and to a lesser extent, in the upper limb girdles and neck [2]. Primary SEF of the visceral organs is exceedingly rare, with only a very few case reports [3]. It has been noted that local recurrences occur in more than 50% of cases, with spread to distant sites in more than 40% of cases. The most common sites of distant metastasis include the lungs (70%), osseous dissemination to multiple bones (41%) and pleura/chest wall (11%) [3]. Poor prognostic factors basically include a proximal site of origin, large tumour size, presence of tumour recurrence and metastases.

The most notable feature of this entity is that it is a histologically low-grade sarcoma with an aggressive clinical course [4]. It is characterised microscopically by low-grade features, namely round cells arranged in a cord like fashion with a stark collagen background almost effacing the tumour cells [4]. Chromosomal abnormalities include 10p11 rearrangement, 12q13 and 12q15 amplification, including the HMGIC transcriptional activator gene [4].

Ultrastructural studies have proven its dense fibroblastic nature replete with a dense collagenous matrix which is indistinguishable from normal osteoid. The closest differential is low-grade fibromyxoid sarcoma (LGFMS). LGFMS is differentiated by a younger age at presentation, indolent course and specific pathological features like bland whorled fibrous areas, myxoid zones and thin-walled vessels [5]. However, a subset of cases may have areas morphologically resembling SEF. Immunohistochemically, there is MUC 4 positivity in both these sarcomas (100% in LGFMS versus 78% in SEF),but molecular studies have shown that LGFMS traditionally has the FUS-CREB3L2 fusion (96%), whereas SEF has the FUS-CREB3L1 fusion [5]. The subset of MUC 4 negative SEF is characterised by YAP1 and KMT2A gene rearrangements [5].

The treatment for SEF is not well defined. For single site disease, surgical excision followed by adjuvant radiotherapy is the modality of choice [6]. In many case series, it has been seen that all radically treated patients underwent tumour relapse, with around 60%–70% experiencing metastatic disease at first relapse. Systemic chemotherapy has dismal outcomes in metastatic disease. In terms of overall chemotherapy responses, similar results in accordance with other types of metastatic soft tissue sarcomas are seen.

## Case discussion

**Figure 1.**
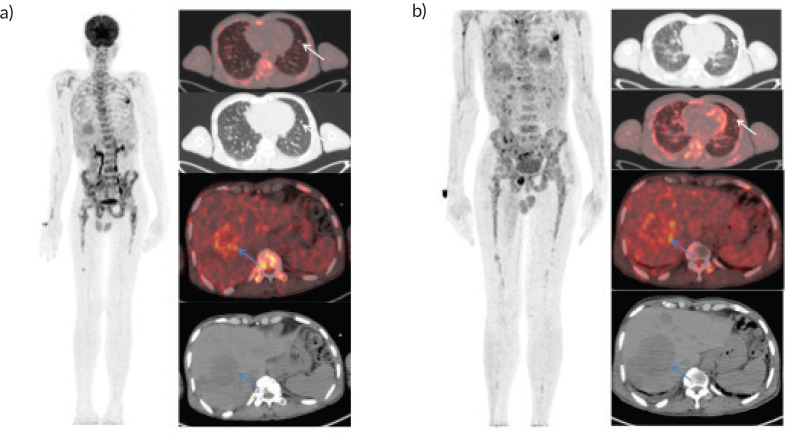
Whole body FDG PET scan. (a): Pretherapy PET/CT shows increased tracer uptake in the thoracic, liver and multiple skeletal regions in maximum intensity projection image (A).Trans-axial fused PET/CT and CT sections shows multiple random distribution parenchymal nodules in the lungs (white arrows) and large necrotic peripheral FDG avid lesion in segment VII/VIII of the liver measuring 5.3 × 5.5 cm .Also noted FDG avid lytic lesions noted in D10 vertebra (blue arrows). (b): Post-therapy PET/CT shows increased tracer uptake in the thoracic, liver and multiple skeletal regions in maximum intensity projection image (A).Trans-axial fused PET/CT and CT sections shows multiple random distribution parenchymal nodules in the lungs(white arrows) and large necrotic peripheral FDG avid lesion in segment VII/VIII of the liver measuring 7.3 × 6.1 cm. Compared to pretherapy PET/CT scan there is increase in size of liver lesions along with increase in number of lung and skeletal lesions suggestive of progressive disease.

**Figure 2.**
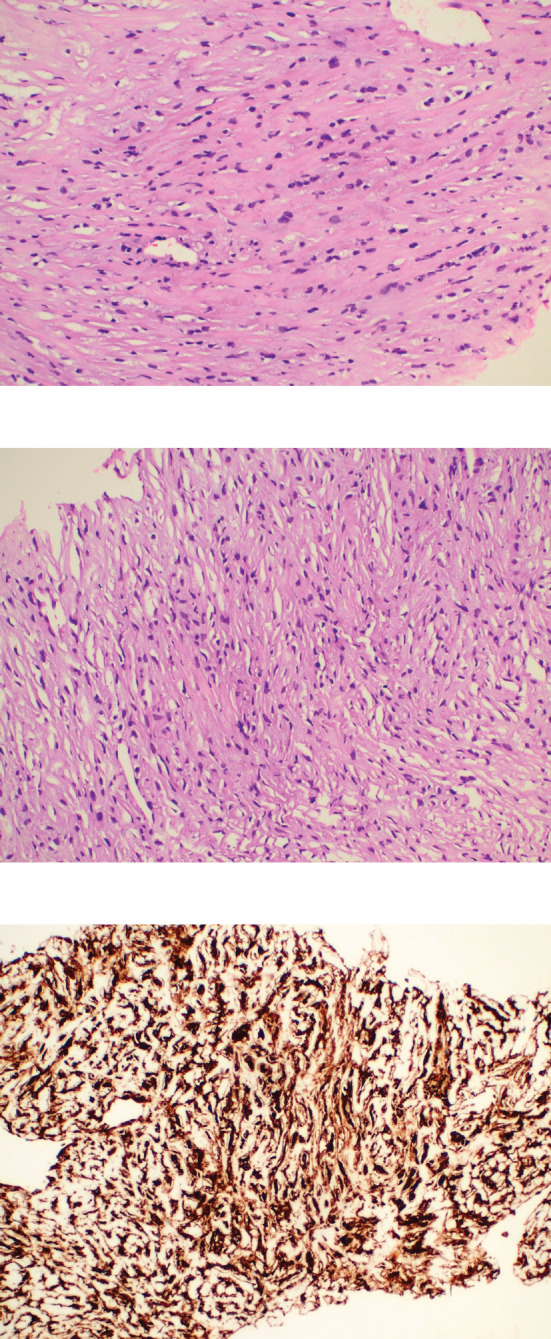
Histopathology images. (a and b): Histological examination of the tumour showing cells arranged predominantly in cords surrounded by dense collagenous stroma. The individual tumour cells exhibit mild to moderate nuclear pleomorphism with finely dispersed chromatin and abundant eosinophilic cytoplasm with characteristic epithelioid morphology. (c): Immunohistochemistry for MUC 4 showing diffuse immunoreactivity in the tumour cells.

A 46-year-old gentleman, presented with low back ache and difficulty in walking for 3 months, associated with loss of appetite and loss of weight. Other notable points in history include a dragging sensation in the right upper abdomen and difficulty in initiating micturition. Clinical examination revealed tenderness in the right upper quadrant of abdomen, and increased tone and decreased power in the right lower limb, with patient being bedridden with an Eastern Co-operative Oncology Group performance status of 4. A baseline positron emission tomography (PET) scan (Figure 1a) revealed lytic and sclerotic lesions with increased fluoro-deoxy glucose (FDG) uptake in the entire axial and appendicular skeleton, multiple subpleural and parenchymal lung nodules and discrete hypodense lesions in segment 7/8 of liver. Keeping in view the patient age and diffuse lytic lesions in PET scan, a multiple myeloma work up was done, including serum electrophoresis and serum free light chain assay, which were all normal. His liver biopsy and bone marrow biopsy showed a poorly differentiated mesenchymal tumour. Despite multiple reviews with sarcoma pathologists; his diagnosis could not be established. Subsequently liver biopsy was repeated and sent again for expert review, which revealed a collagenising spindle cell proliferation comprised of epithelioid and short spindled cells with ovoid nuclei, fine chromatin, inconspicuous nucleoli and scanty eosinophilic cytoplasm (Figure 2a and b). The tumour cells expressed MUC 4 (Figure 2c), while S-100, SOX10, desmin, smooth muscle antigen, CD34 and ETS-related gene were negative, suggestive of SEF. FUS-CREB3L1 fusion was not available in-house, so EWSR1 gene rearrangement by fluorescence in-situ hybridisation (FISH) was done, which was positive. He was started on systemic chemotherapy subsequently. He had disease progression following two lines of therapy, namely cyclophosphamide-doxorubicin (Figure 1b) and gemcitabine-docetaxel. He was later switched to the oral multi-kinase inhibitor pazopanib, following which he had remarkable improvement in his general condition. This patient who was bedridden before therapy, could walk again and there was notable improvement in his abdominal pain, appetite and weight. But the response was short lived, lasting for 2 months, with subsequent PET scans showing disease progression.

## Discussion

The age of our patient is 46 years, which corresponds to the median age in the literature.

Our patient had diffuse skeletal involvement as documented in SEF when compared to LGFMS which has rare bone involvement. The delay in diagnosis of this case was 4 months, which corresponds to the median diagnosis delay in rare soft tissue sarcomas [7]. As SEF is a slowly growing tumour for months or years, majority of the patients present with distant metastasis, as was the case in our patient with multiple lung, liver and osseous metastasis.

In contrast to reported literature where soft tissue primary site was common [3], our patient had primary in the bone, with multiple lytic lesions in the D10 vertebra. The initial bone marrow and liver/spleen involvement led us to work along the lines of haemato-lymphoid malignancy. In the paper by Asakra* et al* [8]*,* SEF can present with diffuse infiltration of the bone marrow, often mimicking carcinoma or leukaemia/lymphoma. Bone marrow involvement is an extremely rare occurrence in sarcomas and is seen mostly in round cell sarcomas or osteosarcomas. EWSR1 break apart FISH may aid in diagnosis, but the fusion partner is CREB3L1/2, unlike in the Ewing’s sarcoma family tumours. Various agents, including single-agent doxorubicin, combination of doxorubicin and ifosfamide, gemcitabine and docetaxel, and trabectedin have been tried, with mixed results [9]. In the paper by Chew *et al* [9]*,* seven out of ten patients with metastatic disease were offered palliative chemotherapy (doxorubicin based), out of which one had partial response, three had stable disease and three had progressive disease, highlighting the poor response of this entity to conventional chemotherapy. Similarly, in our patient, there was no response to any chemotherapy. Recently, immunotherapy has also been tried with encouraging results. A paper by Doshi *et al* [10] describes two cases of metastatic SEF where immunotherapy has been tried. One case had PD-L1 expression of 25% and there was marked regression in restaging imaging following four cycles of nivolumab (3 mg/kg) and ipilimumab (1 mg/kg).

The second case had PD1 expression of 5% and patient showed significant regression of osseous disease and complete resolution of anterior chest wall mass following four cycles of nivolumab and ipilimumab. Similarly, a paper by Monga* et al* [11] describes the use of immunotherapy in metastatic soft tissue sarcomas, wherein one patient with metastatic SEF achieved partial response. In a resource-constrained setting like ours, where immunotherapy is not covered by insurance providers, we chose not to start our patient on nivolumab and ipilimumab because of financial toxicity. Based on the efficacy of pazopanib in non-adipocytic sarcomas as per the PALETTE trial [12], we have started our patient on the same. Even though there are no specific reports of response of SEF to pazopanib, our patient had a response, but it was short lived, and patient progressed after 2 months. Hence, this entity should be noteworthy of the fact that there are hardly any responses to standard chemotherapy and upfront immunotherapy may be considered as the first option.

## Conclusion

Here, we highlight SEF, a rare variety of fibrosing sarcoma, with its distinct epidemiology, clinical presentation and pathological features. We need to understand the overall poor outcome of this disease, its relative chemo-refractoriness and the urgent need for novel therapies.

## Abbreviations

SEF, Sclerosing epithelioid fibrosarcoma; LGFMS, Low-grade fibromyxoid sarcoma; FDG, Fluoro-deoxy glucose; FISH, Fluorescence in-situ hybridisation.

## Authors’ contributions

AS: contributed to conception, acquisition of data, drafted the manuscript, approved submitted version, is personally accountable for own contribution

JM and AB: helped with the histopathology images and interpretation

SAS: contributed with PET scan interpretation and response assessment

SR: contributed to conception, design, acquisition, data interpretation and approved the submitted study, agreed to be personally accountable for own contribution.

## Funding

No funding has been received for this study.

## Availability of data and materials

All data generated are included in the published manuscript.

## Declarations

### Ethical approval and consent to participate

Not applicable.

### Consent for publication

Written informed consent was obtained from the patient for publication of this case report and any accompanying images.

## Conflicts of interest

None.

## References

[1] Smith PJ, Almeida B, Krajacevic J (2008). Sclerosing epithelioid fibrosarcoma as a rare cause of ascites in a young man: a case report. J Med Case Rep.

[2] Chow LTC, Lui YH, Kumta SM (2004). Primary sclerosing epithelioid fibrosarcoma of the sacrum: a case report and review of the literature. J Clin Pathol.

[3] Khezami K, Gharbi A, Chabaane M (2021). Primary sclerosing epithelioid fibrosarcoma presenting as lombosciatic syndrome: case report and literature review. Int J Surg Case Rep.

[4] Ossendorf C, Studer GM, Bode B (2008). Sclerosing epithelioid fibrosarcoma: case presentation and a systematic review. Clin Orthop Relat Res.

[5] Antonescu CR (2002). Sclerosing epithelioid fibrosarcoma. Pathol Case Rev.

[6] Shankar DS, Phan N, Shirsat H (2020). A 27-year-old female with sclerosing epithelioid fibrosarcoma of the left ethmoid sinus. Otolaryngol Case Rep.

[7] Brouns F, Stas M, De Wever I (2003). Delay in diagnosis of soft tissue sarcomas. Eur J Surg Oncol.

[8] Asakra R, Zaidi S, Thway K (2017). Metastatic sclerosing epithelioid fibrosarcoma in bone marrow. Int J Surg Pathol.

[9] Chew W, Benson C, Thway K (2018). Clinical characteristics and efficacy of chemotherapy in sclerosing epithelioid fibrosarcoma. Med Oncol.

[10] Doshi SD, Oza J, Remotti H (2021). Clinical benefit from immune checkpoint blockade in sclerosing epithelioid fibrosarcoma: a translocation-associated sarcoma. JCO Precis Oncol.

[11] Monga V, Skubitz KM, Maliske S (2020). A retrospective analysis of the efficacy of immunotherapy in metastatic soft-tissue sarcomas. Cancers.

[12] Van Der Graaf WT, Blay J, Chawla SP (2011). PALETTE: a randomized, double-blind, phase III trial of pazopanib versus placebo in patients (pts) with soft-tissue sarcoma (STS) whose disease has progressed during or following prior chemotherapy – an EORTC STBSG Global Network Study (EORTC 62072). J Clin Oncol.

